# The epiphytic microbiota of sour rot-affected grapes differs minimally from that of healthy grapes, indicating causal organisms are already present on healthy berries

**DOI:** 10.1371/journal.pone.0211378

**Published:** 2019-03-27

**Authors:** Megan E. Hall, Isabelle O’Bryon, Wayne F. Wilcox, Michael V. Osier, Lance Cadle-Davidson

**Affiliations:** 1 Section of Plant Pathology and Plant-Microbe Biology, School of Integrative Plant Science, Cornell University, NYS Agricultural Experiment Station, Geneva, New York, United States of America; 2 Thomas H. Gosnell School of Life Sciences, Rochester Institute of Technology, Rochester, New York, United States of America; 3 United States Department of Agriculture–Agricultural Research Service, Grape Genetics Research Unit, Geneva, New York, United States of America; University of California Davis, UNITED STATES

## Abstract

Sour rot is a disease complex produced by an interaction between grape berries and various species of yeast and acetic acid bacteria in the presence of *Drosophila* fruit flies. While yeast and bacteria are consistently found on healthy grape berries worldwide, we explored whether the composition of these epiphytic communities differed depending on the presence or absence of sour rot symptoms. Using high-throughput sequencing, we characterized the microbiome of sour rot-affected grapes from two geographical areas across two years. In 2015 and 2016, both healthy and sour rot-affected berries were collected from commercial and research vineyards in Geneva, NY and commercial vineyards in Tasmania, AUS. In this experiment, all associated organisms grouped together primarily by location, and not by presence/absence of symptoms or cultivar. The predominant difference between asymptomatic and symptomatic samples, regardless of location, was the abundance of *Acetobacter* species, which were significantly more plentiful in the symptomatic samples. Yeast genera such as *Candida*, *Hanseniaspora*, *Pichia* and *Saccharomyces* were abundant in both sets of samples, but varied by region. The consistent presence of yeast species and the increased abundance of acetic acid-generating bacteria is consistent with our understanding of their etiological role in sour rot development. In 2016, diseased grapes also were collected from vineyards in Fredonia, NY, and Modesto, CA. Consistent with our comparison study, all associated organisms again grouped together primarily by location. Yeast genera such as *Candida*, *Hanseniaspora*, *Pichia* and *Saccharomyces* were abundant in both sets of samples, but varied by region. The consistent presence of yeast species and the abundance of acetic acid-generating bacteria in both experiments is consistent with our understanding of their etiological role in sour rot development.

## Introduction

The surface of a healthy grape berry is the site of abundant populations of yeast and bacteria. While the microbial composition of these dynamic communities varies by grape cultivar, site and sampling time [1–4], there are also significant consistencies across regions. Grape-associated microbes have been studied by macerating grapes [2,5] or sampling grape musts after harvest [1,6,3], and the research has repeatedly shown the presence of various yeast species and members of the bacterial orders Bacillales, Enterobacteriales, Pseudomonadales and Rhodospirillales.

While the ecology of the healthy grape at harvest has been well researched in recent years, changes in the microbial populations due to disease are under-characterized, especially in relation to sour rot. Recent research indicates that development of grape sour rot requires the concomitant involvement of yeast, bacteria and *Drosophila* fruit flies [7]. The yeast ferment the sugars in the grape pulp, producing ethanol, and after wounding, the newly aerobic environment allows the bacteria to oxidize the ethanol into acetic acid, generating the symptomatic sour aroma. However, it is not known how microbial populations on the surface of diseased grapes differ from those on the surface of healthy grapes at harvest. Several yeast species have been shown to cause sour rot symptoms when co-inoculated with acetic acid bacteria in the presence of *Drosophila* [7]. However, little is known about whether the species involved differ by region or vary in abundance between the surface of symptomatic and asymptomatic grape berries. Examining whether there are patterns within these microbial populations will contribute to our understanding and management of the sour rot disease complex.

Metagenomic analysis of grape berry surfaces is complicated by the small epiphytic biomass, which may be tightly linked with the waxy berry cuticle. While most grape metagenomic studies, including those cited above, have homogenized plant tissues prior to DNA isolation, one of our objectives in the present study was to investigate the epiphytic DNA associated with healthy and sour rot-infected samples, isolated directly from whole grape berries. Our overarching goal was to identify the taxa associated with the presence and absence of sour rot symptoms and to characterize differences between these microbiomes. To address this goal, two experiments were conducted to look at epiphytic microbial communities using Illumina sequencing methods: (i) healthy versus sour rot-affected whole berries were collected from commercial and research vineyards in Geneva, NY and commercial vineyards in Tasmania, AUS and compared; (ii) sour rot-infected samples were collected from commercial vineyards in Modesto, California and Fredonia, New York and the community composition was characterized.

## Materials and methods

### Grape sampling for analysis of epiphytic microbes

Shortly before harvest in 2015, grapes were collected from three privately-owned commercial vineyards and one Cornell University research vineyard in the Finger Lakes region of New York (42°52′N; 76°59′W) (Table 1). Shortly before harvest in 2016, grapes were collected from eight privately-owned commercial vineyard blocks in Tasmania, Australia (41°45’S; 145°97’E) (Table 1). Permission to enter each one of the privately-owned vineyard blocks was obtained through communication with the vineyard owner or vineyard manager. In every vineyard block, 12 panels were randomly selected, and one cluster exhibiting sour rot symptoms was selected from each panels. To account for potential spatial variability within a cluster, three asymptomatic berries, located at the (i) tip of the cluster, (ii) anterior side (toward canopy exterior) and (iii) posterior side (toward canopy interior), were cut from each cluster above the pedicel attachment using scissors that were immersed in 95% ethanol between samples, and all three berries were dropped directly into an individual 50-mL Falcon tube containing 5 ml of a TE buffer solution (10mM Tris-HCl+1mM EDTA, ph 8.0) with 10% w/v NaCl. The same procedure was used with three symptomatic berries from the same clusters. Tubes were immediately sealed and placed in a Styrofoam cooler containing an ice pack for transport to the laboratory for DNA extraction.

**Table 1:** Number of samples, percent passing quality filtering and OTU assignment by phenology, year, and Kingdom and total OTU abundance for all samples collected.

	Epiphytic				Whole berry			
n	2015 Finger Lakes 96		2016 Tasmania 192		Modesto, CA 54		Fredonia, NY 36	
	Fungi	Bacteria	Fungi	Bacteria	Fungi	Bacteria	Fungi	Bacteria
Filtered number	51	39	130	75	54	54	36	36
Percent of total	53.1	40.1	67.7	39.1	100	100.0	100	100

### Grape sampling for analysis of whole-berry, culturable microbes

Sour rot-affected clusters from commercial vineyards in Fredonia, NY (42°26’N;79°17’W) and Modesto, CA (37°38’N;120°59’W) were randomly selected from six vines of *Vitis* interspecific hybrids at each location (cvs. Brianna, Valiant, Frontenac, Fredonia, LaCrosse and Marquis in Fredonia, NY; and unnamed breeding lines in Modesto, CA). Sample collection at both privately-owned vineyards was performed by employees of these commercial operations. The infected clusters from each cultivar were placed in polyethylene bags, put in a cooler containing an ice pack and transported to the laboratory in Geneva, NY. In the laboratory, three asymptomatic berries (representing the tip and two opposite sides) were removed from the cluster above the pedicel attachment using surface-sterilized scissors, as described above. The berries were macerated in the polyethylene sample bags, and 100 μl of juice was pipetted onto three plates each of Yeast Peptone Dextrose (YPD) and Mannitol agars. The plates were incubated at 24°C for 3 days. After 3 days of growth, 1 ml of sterile distilled water was pipetted onto each plate, and the cells were disrupted using a sterile L-shaped cell spreader (Fisher Scientific, Pittsburgh, PA). This suspension was then pipetted into a 50-ml Falcon tube containing 5 ml of TE buffer with 10% NaCl and frozen at -4°C until further processing.

### DNA extraction

To each sample in the TE-NaCl solution, 500 μl of 10% SDS was added, vortexed for 5 s and left at room temperature for 15 min. A freeze-thaw sequence consisting of 30 min in a -80°C freezer and 5 min in a 60°C water bath was repeated three times to lyse the fungal and bacterial cells. A 750-μl aliquot of the solution was transferred to a 2 ml microfuge tube, along with 750 μl ice-cold isopropanol. The solution was centrifuged for 10 min at 9600 *g*. The supernatant was carefully transferred to a new microfuge tube, 500 μl ice-cold 95% ethanol was added, and this was centrifuged at 9600 *g* for 1 min. After removing the supernatant by pipet, the pellet was re-suspended in 100 μl TE buffer and the recovered DNA was then stored at 4°C until further use.

### Amplification and sequencing

Genomic DNA was sent to the Cornell University Sequencing facility in Ithaca, NY for sequencing library preparation and 2x250bp paired-end sequencing on an Illumina MiSeq sequencer (Illumina, San Diego, CA, USA). Dual-barcoded Nextera library preparation followed AmpSeq protocols [8] but with singleplex PCR. The V4 domain of bacterial 16S rRNA was amplified using primers (all sequences shown 5′ to 3′): F515 (GTGTGCCAGCMGCCGCGGTAA) and R806 (GGACTACHVGGGTWTCTAAT). Fungal internal transcribed spacer (ITS) 1 loci were amplified using primers BITS (CTACCTGCGGARGGATCA) and B58S3 (GAGATCCRTTGYTRAAAGTT) [1]. To enable sample barcoding, AmpSeq linkers were added to the 5′ end of each locus-specific primer. As detailed in Yang et al., 2016, the linker to accommodate S5xx barcodes for each forward primer is: TCGTCGGCAGCGTCAGATGTGTATAAGAGACAG. The linker to accommodate N7xx barcodes for each reverse primer is: GTCTCGTGGGCTCGGAGATGTGTATAAGAGACAG.

### Bioinformatic analysis

For pre-processing barcode-sorted raw read data in QIIME [9], multiple_extract_barcodes was executed on two folders, containing R1 and R2 reads; file names were changed to allow QIIME to correctly identify the specifiers (_barcode, _map, _R1, _R2); and mapping files were created and formatted according to standard protocols in QIIME. To combine the demultiplexed files into one file, multiple_split_libraries_fastq was executed using two directories containing all R1 or all R2 fastq files and their corresponding mapping and barcode files with the following parameters: mapping extension set to txt, the demultiplexing method was mapping_barcode_files, and the read, barcode, sample ID, and mapping indicators were R[1/2].fastq, _barcodes.fastq, ‘.’, and _map.txt, respectively. Multiple_split_libraries_fastq calls split_libraries_fastq, which was given the following parameters: barcode type was 17, phred offset was 33, phred quality threshold was 20, maximum bad run length was 300, and minimum per read length fraction was .01.

To assign the bacterial sequences to OTUs, pick_closed_reference_otus [10] was executed, the seqs.fastq file with assign taxonomy and reverse strand match enabled, and RDP maximum memory set to 60000. For reference sequences, Greengenes 13_8 97_otu_taxonomy.txt and 97_otus.fasta files were used [11–12]. The otu_table_mc2.biom files from the R1 and R2 reads were then merged using QIIME’s merge_otu_tables. To determine fungal taxonomies, pick_open_reference_otus was executed with the reference file path, the template file path, and reference sequence file path were all set to the UNITE 97% file sh_refs_QIIME_ver7_97_28.06.2017.fasta and the ID to taxonomy file path was set to the UNITE file called sh_taxonomy_QIIME_ver7_97_28.06.2017.txt. Reverse strand match and suppress lane mask filter were set to true, the assignment method was set to blast, RDP maximum memory set to 60000, and the entropy threshold was set to 0.10. Otu_table_mc2_w_tax.biom files from the R1 and R2 reads were then merged using QIIME’s merge_otu_tables.

Rare OTUs were removed by filtering if they had less than 0.0001% of the total abundance from within that biom file. Biom files were converted into spf files using the biom_to_stamp.py script provided by STAMP. The original mapping file and the spf file were read into STAMP, and an ANOVA test was done using the Tukey-Kramer method set to 0.95 and a *P* value filter of 0.05. The percentage of each taxon in each sample was calculated. Then the mean of the percentages for each taxon within each treatment was calculated and plotted in R. Organisms that could not be identified to the family level were excluded from the analysis.

Heatmaps were made in R v.3.3.2 using the pheatmap package [13–14]. The colors represent the log of the relative mean frequency for each taxon. If a taxon was not seen in a given group the value was assigned to the lowest value in the matrix. Hierarchical clustering was done using the complete method, the rows were clustered using the Euclidean method, and the columns were clustered using the Manhattan method.

## Results

Across all analyses, 99.7% of the bacterial organisms and 99.5% of fungal organisms that could be identified to the Kingdom level were successfully identified to genus, so a minimal number of reads were excluded from the analyses.

Two-thirds of field samples collected in Tasmania returned fungal OTUs compared to one-half from Finger Lakes vineyards and both sites returned the same 40% of bacterial OTUs (Table 1). All of the samples from Modesto, CA and Fredonia, NY, which were cultured in the lab before sequencing, returned both fungal and bacterial OTUs.

**Fig 1:**
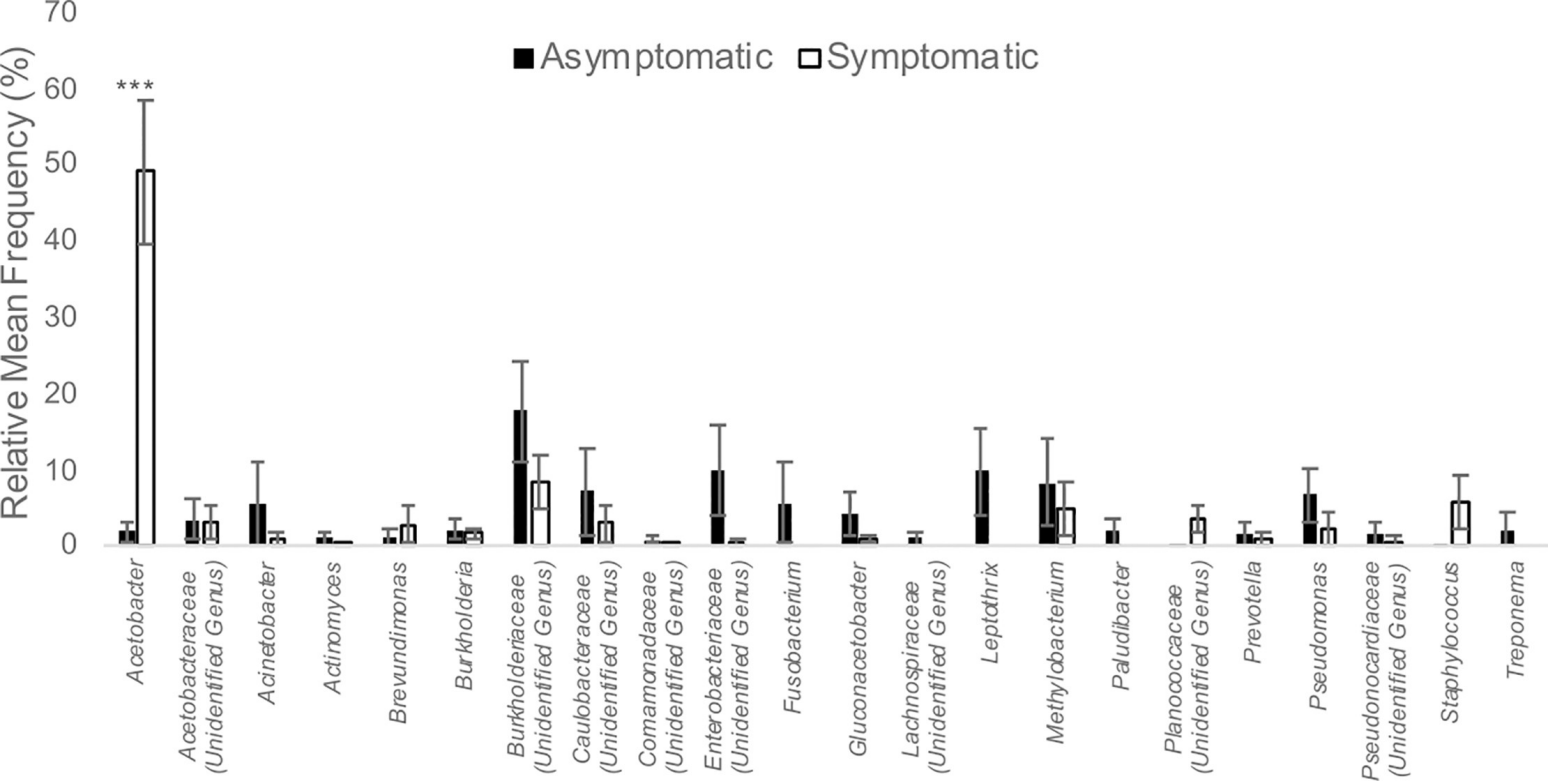
Bacterial OTUs in asymptomatic and symptomatic sour rot samples in the Finger Lakes. The relative mean frequency (%) of bacterial OTUs represented in 18 asymptomatic and 21 symptomatic sour rot samples from three commercial vineyards of *Vitis vinifera* cv. Riesling and Pinot Gris and one research vineyard of *Vitis* interspecific hybrid cv. Vignoles in the Finger Lakes region of New York in 2015. Asterisks (*) above a pair of bars denote a statistically significant difference, as determined by a two-sided t-test: *** *P* < 0.001.

**Fig 2:**
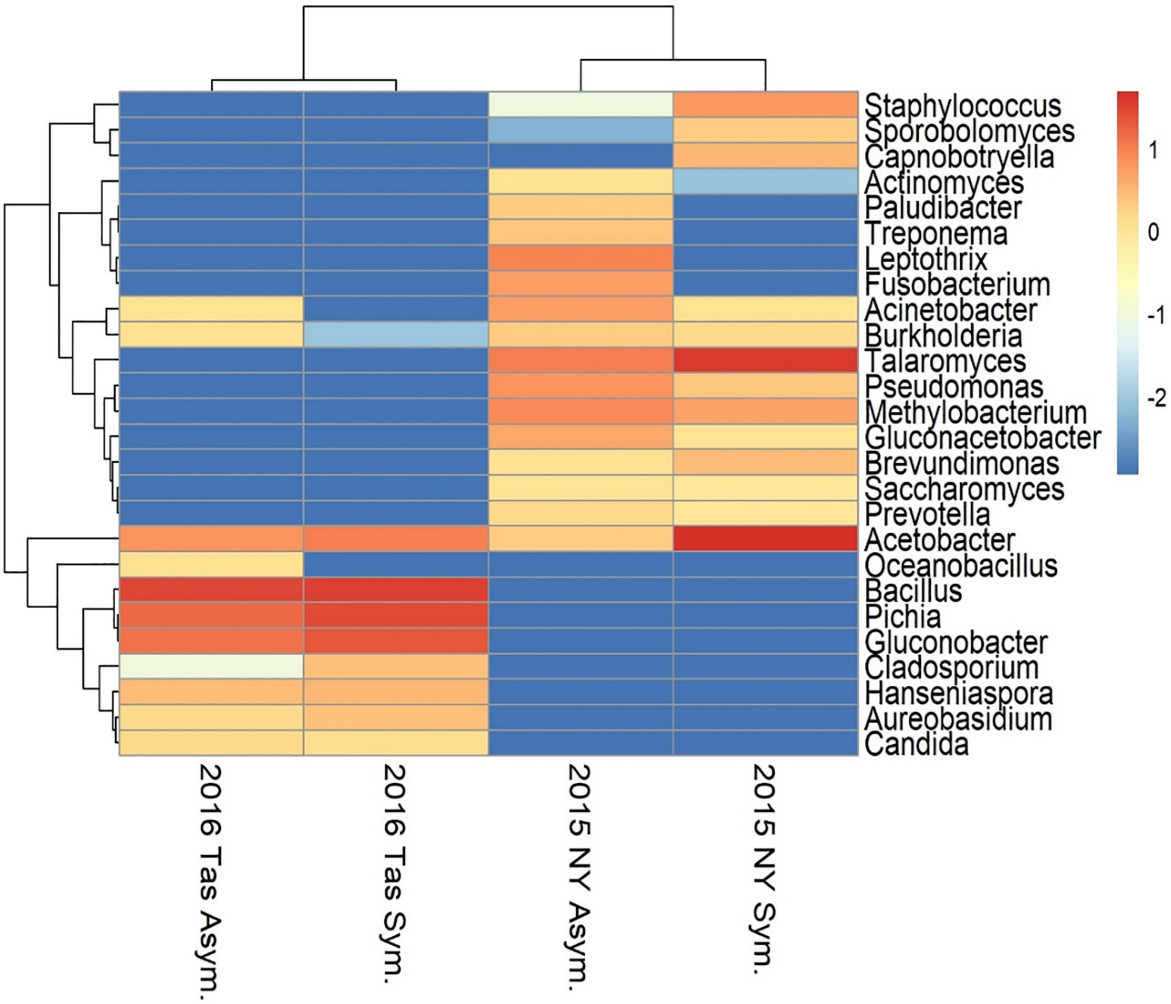
Heatmap representing the log of the relative mean frequency for each taxon. Colors indicate frequency (blue = low relative mean frequency; red = high relative mean frequency). Hierarchical clustering was done using the complete method, the rows were clustered using the Euclidean method, and the columns were clustered using the Manhattan method.

**Fig 3:**
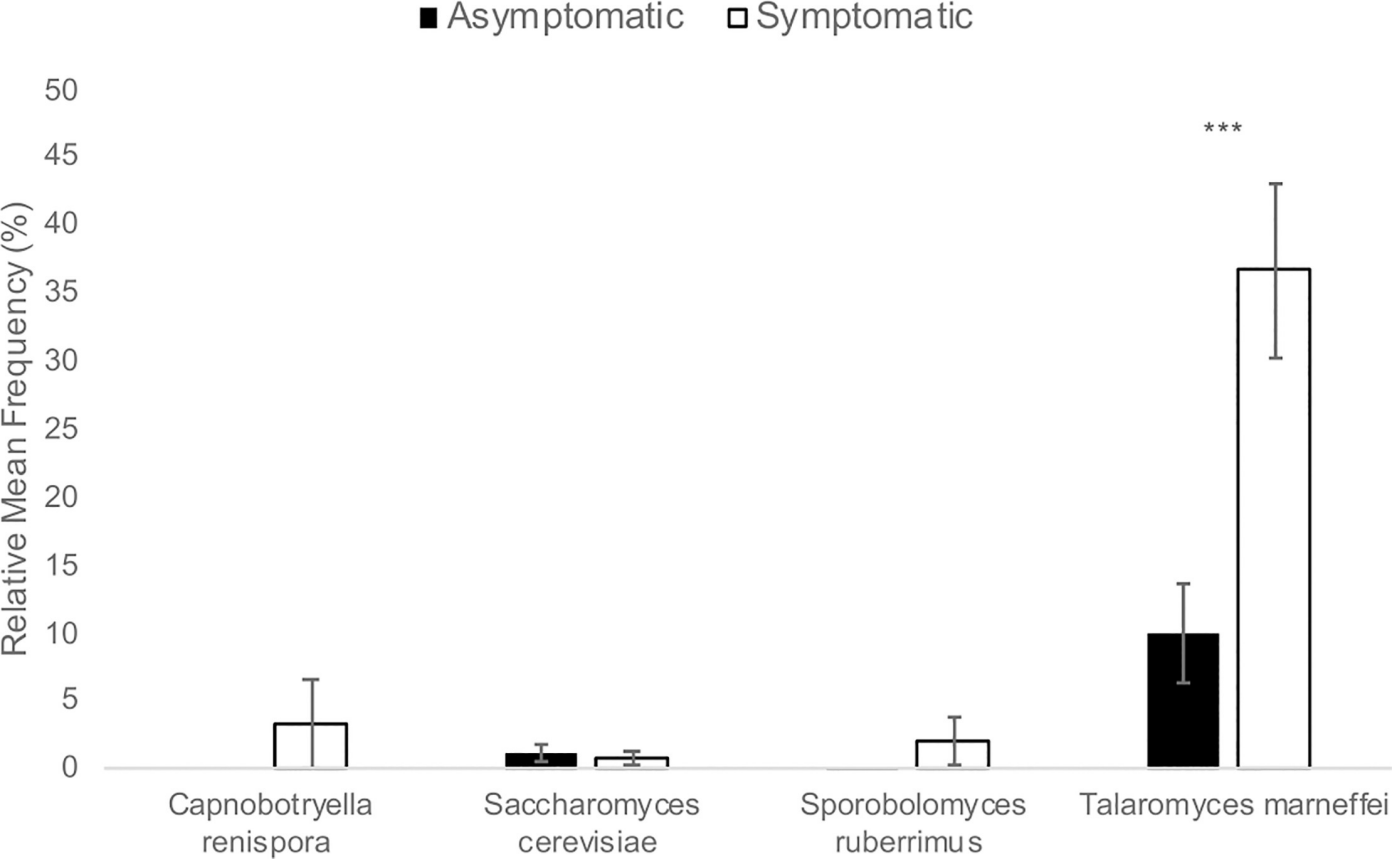
Fungal OTUs in asymptomatic and symptomatic sour rot samples in the Finger Lakes. The relative mean frequency (%) of fungal OTUs represented in 22 asymptomatic and 29 symptomatic sour rot samples from two commercial vineyards of Vitis vinifera cv. Riesling and Pinot Gris and one research vineyard of Vitis interspecific hybrid cv. Vignoles in the Finger Lakes region of New York in 2015. Asterisks (*) above a pair of bars denote a statistically significant difference, as determined by a two-sided t-test: *** *P* < 0.001.

Finger Lakes grape berries collected in 2015 had similar bacterial and fungal microbiota, regardless of the presence of sour rot symptoms (Figs 1 and 2). However, 20 bacterial genera were detected on asymptomatic berries versus 12 on symptomatic berries (Fig 2). While the differences in relative mean frequency of OTU detection were non-significant for most genera represented, *Acetobacter* was 24-fold enriched on symptomatic versus asymptomatic berries (*P* < 0.0001; Figs 1 and 2). Manhattan method clustering grouped the organisms by location, not by presence/absence of symptoms, except for *Acetobacter* which was present in all samples, and had higher frequencies in the symptomatic samples than the asymptomatic samples (Fig 2). For fungi, the only significant difference between the asymptomatic and symptomatic samples was the presence of the filamentous ascomycete *Taloromyces marneffei*, which was 3.7-fold enriched on symptomatic versus asymptomatic berries (*P* < 0.01, data not shown), and present only in the New York samples (Fig 3). *Saccharomyces cerevisiae* represented approximately 1% of the OTUs in both asymptomatic and symptomatic samples.

**Fig 4:**
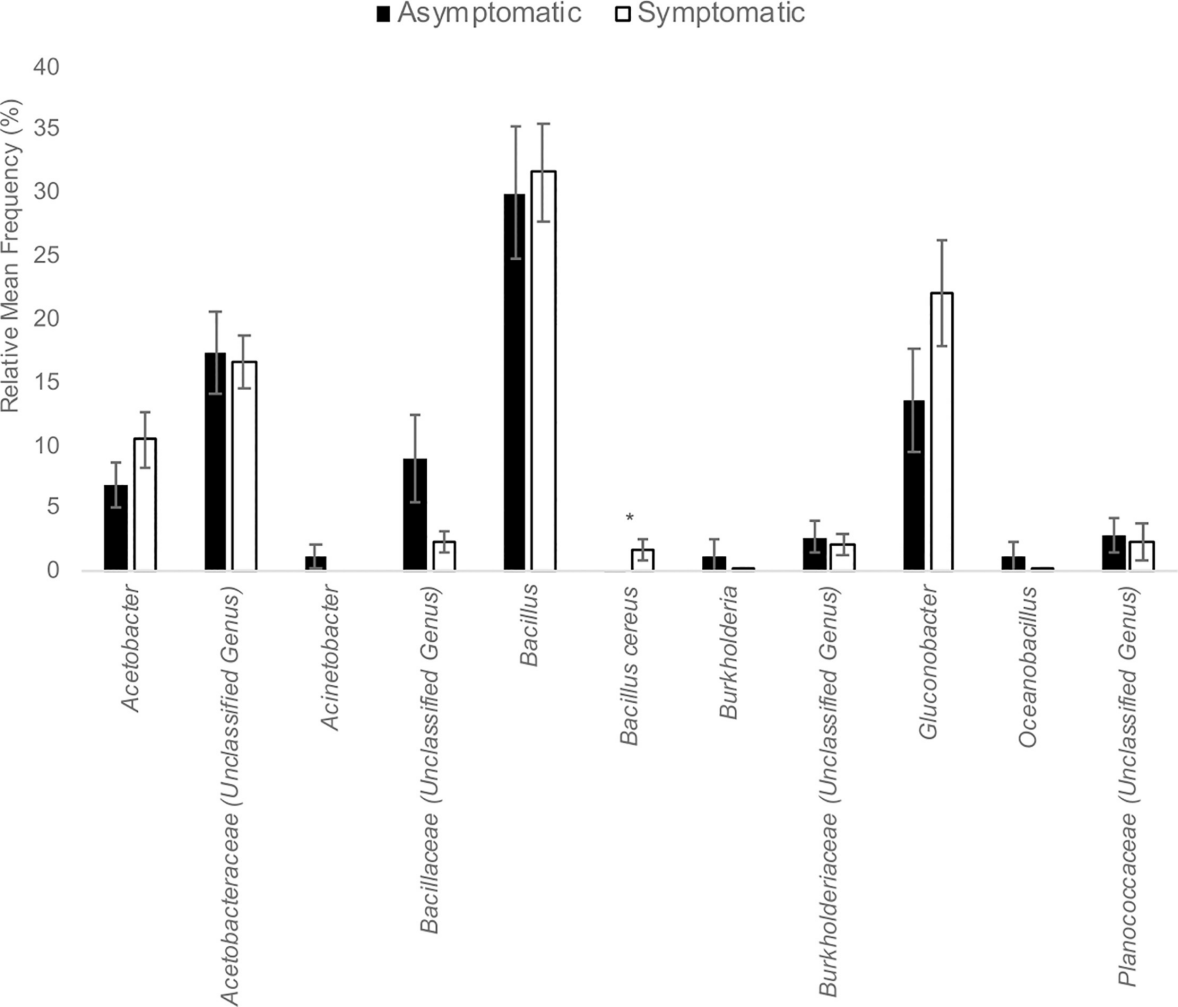
Bacterial OTUs in asymptomatic and symptomatic sour rot samples in the Tasmania. The relative mean frequency (%) of bacterial OTUs represented in 41 asymptomatic and 34 symptomatic sour rot samples from two commercial vineyards of *V*. *vinifera* cv. Riesling and two commercial vineyards of *V*. *vinifera* cv. Pinot Noir in Tasmania, Australia in 2016. Asterisks (*) above a pair of bars denote a statistically significant difference, as determined by a two-sided t-test: * *P* < 0.05.

**Fig 5:**
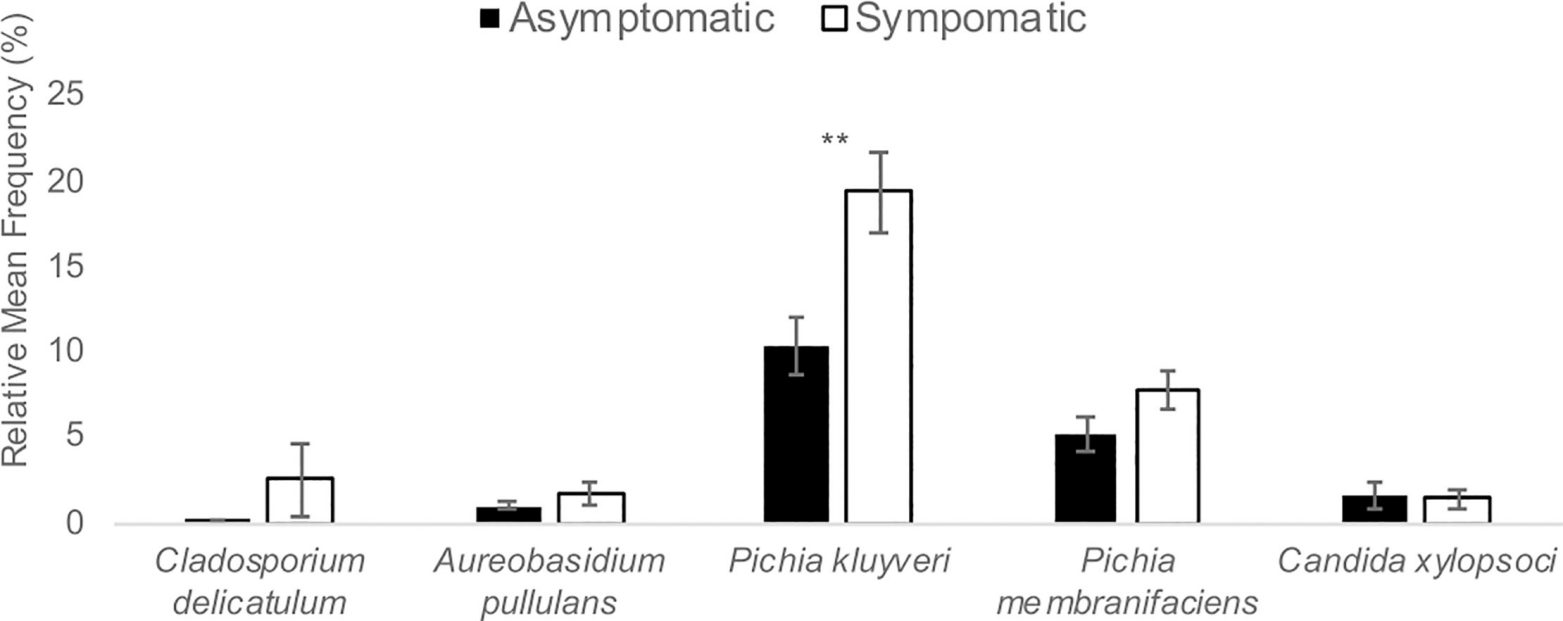
Fungal OTUs in asymptomatic and symptomatic sour rot samples in the Tasmania. The relative mean frequency (%) of fungal OTUs represented in 86 asymptomatic and 44 symptomatic sour rot samples from two commercial vineyards of *V*. *vinifera* cv. Riesling and two commercial vineyards of *V*. *vinifera* cv. Pinot Noir in Tasmania, Australia in 2016. Asterisks (*) above a pair of bars denote a statistically significant difference, as determined by a two-sided t-test: ** *P* < 0.01.

As with the 2015 Finger Lakes samples, there were few differences in the bacterial and fungal microbiota between symptomatic and asymptomatic grape berries collected from Tasmania in 2016 (Fig 4). Again, more bacterial genera were detected on asymptomatic berries (10) than on symptomatic berries (8). While the relative mean frequency was non-significant for most OTUs represented, *Bacillus cereus*, a species detected only in the Tasmanian samples (Fig 2), was 17-fold enriched on symptomatic versus asymptomatic berries (*P* = 0.03; Fig 4). The family Acetobacteraceae was common on both asymptomatic and symptomatic berries (20.4 and 32.5% of the OTUs, respectively), and in both locations (Figs 1 and 2). The genus *Acetobacter*, which was highly enriched on sour rot-affected berries in the Finger Lakes, was equally common on symptomatic and asymptomatic berries in Tasmania (10.4 and 6.9% of the OTUs, respectively; *P* = 0.22) (Fig 1). For fungal OTUs, *Pichia kluyveri* and *P*. *membranifaciens* composed the majority of the OTUs from the Tasmanian samples (Fig 5). *Pichia kluyveri* was 2-fold enriched on symptomatic versus asymptomatic berries (*P* < 0.01). Similar to the 2015 Finger Lakes samples, OTUs were common from more fungal taxa on asymptomatic berries than on symptomatic berries (five versus three, respectively) (Fig 5). The presence of yeast and *Acetobacter* was consistent across the asymptomatic and symptomatic berries collected in both 2015 and 2016, albeit with different frequencies, as was the abundance of species.

On symptomatic berries collected in Modesto, CA, 22 fungal and bacterial groups were represented following culture, with the majority of the reads from Bacillales (63.7% of bacterial OTUs) and Saccharomycetales (52% of fungal OTUs) (Table 1). On symptomatic berries collected in Fredonia, New York, 19 groups were represented following culture, and the majority of the reads came from *Pseudomonas* spp. (54.5% of bacterial OTUs), Acetobacteraceae (29.6% of bacterial OTUs), and Saccharomycetales (64.5% of fungal OTUs) (Table 2). Organisms in the families Acetobacteraceae and Enterobacteraceae, along with *Aureobasidium pullulans*, *Metschnikowia* spp. and *Pichia* spp. were expressed in both Modesto and Fredonia sample sets at a rate of more than 1% (Table 3).

**Table 2:** The relative mean frequency (%) of bacterial and fungal OTUs represented in 54 sour rot-affected samples from six *Vitis* interspecific hybrid crosses in Modesto, CA in 2016.

OTU	%
Bacteria	
*Acetobacteraceae (Unclassified Genus)*	3.5
*Bacillaceae* (Unclassified Genus)	16.5
*Bacillales* (Unclassified Family)	3.6
*Bacillus*	33.7
*Bacillus cereus*	1.7
*Bacillus flexus*	8.2
*Brachybacterium*	4.4
*Brachybacterium conglomeratum*	5.0
*Burkholderiaceae* (Unclassified Genus)	6.4
*Enterobacteriaceae* (Unclassified Genus)	7.2
*Gluconobacter*	3.9
*Serratia*	5.9
Fungi	
*Aureobasidium pullulans*	4.0
*Penicillium polonicum*	4.9
*Penicillium vanderhammenii*	6.4
*Metschnikowia chrysoperlae*	16.2
*Metschnikowia pulcherrima*	15.8
*Pichia kluyveri*	10.9
*Pichia membranifaciens*	2.6
*Saccharomyces cerevisiae*	6.6
*Rhodosporidiobolus colostri*	23.0
*Tsuchiyaea wingfieldii*	9.7

**Table 3:** The relative mean frequency (%) of bacterial and fungal OTUs represented in 36 sour rot-infected samples from *Vitis* interspecific hybrid cvs. Brianna, Valiant, Frontenac, Fredonia, LaCrosse and Marquis in Fredonia, NY.

OTU	%
Bacteria	
*Acetobacter*	3.2
*Acetobacteraceae (Unclassified Genus)*	4.1
*Enterobacteriaceae (Unclassified Genus)*	5.5
*Gluconobacter*	22.3
*Leuconostoc*	1.3
*Leuconostocaceae (Unclassified Genus)*	1.1
*Pseudomonas viridiflava*	3.3
*Pseudomonas*	51.2
*Serratia*	3.8
*Stenotrophomonas*	1.2
*Xanthomonadaceae (Unclassified Genus)*	2.9
Fungi	
*Aureobasidium pullulans*	4.4
*Botrytis caroliniana*	18.0
*Metschnikowia chrysoperlae*	4.8
*Metschnikowia pulcherrima*	4.1
*Pichia kluyveri*	3.7
*Pichia membranifaciens*	48.0
*Pichia terricola*	3.9
*Papiliotrema flavescens*	13.1

## Discussion

Recent research into the etiology of sour rot has shown that the interactive involvement of yeast, bacteria and *Drosophila* is necessary for symptom development (Hall et. al. 2018, Hall 2018), yet the dynamics of the microbial system that brings about those symptoms are still unknown. Although it was already known that there is an abundance of yeast and bacteria on healthy grapes, we sought to understand whether those microbial populations changed when sour rot symptoms developed.

The changes in yeast and bacterial populations that we documented in four different regions illustrates the dynamics of the grape surface microbiota associated with sour rot development. Many yeast species in the presence of acetic acid bacteria can cause sour rot symptoms (Hall et. al. 2018; Hall 2018). This present comparison of the microbiota of healthy and sour rot-affected samples from multiple regions demonstrates that a range of yeast are present on the grape surface, which has been shown in previous regional microbial studies comparing healthy grapes (Bokulich et. al. 2014; Pinto et. al. 2015; Setati et. al. 2015; Mezzasalma et. al. 2017; Zarraonaindia et. al. 2015). While various microbes were present in different frequencies depending on the region, many of the same organisms were prevalent in every sample, regardless of symptomology, such as *P*. *kluyveri*, *P*. *membranifaciens*, *S*. *cerevisiae*, *M*. *chrysoperlae* and *M*. *pulcherrima*. The ubiquity of acetic acid bacteria genera, either *Acetobacter* or *Gluconobacter*, is also consistent with our research into causal organisms of sour rot, as referenced above. The increase in abundance of bacterial genera such as *Pseudomonas* in the Fredonia infected samples and *Bacillus* in the Modesto infected samples, could possibly be the result of secondary colonizers benefiting from necrosis of the grape berries and leakage of their contents. A similar effect could be occurring with the increased abundance of *Talaromyces marneffei* in the 2015 New York diseased samples. Additionally, bacterial diversity was lower diseased samples, while acetic acid bacteria became more prevalent, indicating that the increased abundance of AAB could be indicative of it outcompeting other bacteria on the diseased berry surface.

Another consideration is that these measurements were taken at just one moment in time; they do not represent the microbial changes that occur during the disease progression. If we were to examine the surface microbiota throughout symptom development, we may see the yeast populations change as ethanol accumulates within the grape berries, e.g., changing from a higher abundance of relatively-intolerant *Pichia* species to higher populations of more-tolerant *Saccharomyces* species. A similar situation may develop for bacterial genera as acetic acid accumulates.

Extracting DNA from the grape berry surface presented us with a challenge due to the low amount of DNA on the grape surface as well as the difficulty of extracting it from the surface because of the berries’ waxy cuticle. While some researchers have used commercial kits to extract this low quantity of DNA off the grape surface in the laboratory (Zarraonaindia et. al. 2015), we sought to maximize the amount of DNA while limiting contamination by cutting berries directly into a high-salt buffer solution in the field, as the first step of the DNA extraction process. Because the amount of DNA that we successfully extracted was sometimes low, we followed a standard practice to represent our results through relative mean frequencies of various organisms within the samples (Caporaso et al. 2010), but this DNA extraction process allowed us to compare these two unique microbial communities.

We found a consistent presence of yeast species, acetic acid bacteria and members of the Enterobacteriaceae family, as other researchers have previously noted (Bokulich 2014 and 2016; Pinto 2015), but the shifts that occurred within these populations after sour rot developed suggest that the same organisms present on the surface of healthy berries are the ones also associated with disease symptoms. This presents an interesting question about how controlling sour rot-associated microbes in the field could affect the microbial identity, or terroir, of the resulting wines. Our understanding of the sour rot complex is still evolving, but comparing the dynamics of the microbial communities on healthy and diseased grapes demonstrates that grape ecology varies significantly based on geography, and that causal organisms are part of the ecology of the healthy grape. This suggests that there are non-microbial factors involved in symptom development, such as the presence of *Drosophila* and wounding of the grape berry.
